# Identification of Genomic Alterations in Pancreatic Cancer Using Array-Based Comparative Genomic Hybridization

**DOI:** 10.1371/journal.pone.0114616

**Published:** 2014-12-11

**Authors:** Jian-Wei Liang, Zhi-Zhou Shi, Tian-Yun Shen, Xu Che, Zheng Wang, Su-Sheng Shi, Xin Xu, Yan Cai, Ping Zhao, Cheng-Feng Wang, Zhi-Xiang Zhou, Ming-Rong Wang

**Affiliations:** 1 Department of Abdominal Surgical Oncology, Cancer Hospital and Institute, Chinese Academy of Medical Sciences, Peking Union Medical College, Beijing, China; 2 Faculty of Medicine, Kunming University of Science and Technology, Kunming, China; 3 Department of Pathology, Cancer Hospital and Institute, Chinese Academy of Medical Sciences, Peking Union Medical College, Beijing, China; 4 State Key Laboratory of Molecular Oncology, Cancer Hospital and Institute, Chinese Academy of Medical Sciences, Peking Union Medical College, Beijing, China; The University of Hong Kong, China

## Abstract

**Background:**

Genomic aberration is a common feature of human cancers and also is one of the basic mechanisms that lead to overexpression of oncogenes and underexpression of tumor suppressor genes. Our study aims to identify frequent genomic changes in pancreatic cancer.

**Materials and Methods:**

We used array comparative genomic hybridization (array CGH) to identify recurrent genomic alterations and validated the protein expression of selected genes by immunohistochemistry.

**Results:**

Sixteen gains and thirty-two losses occurred in more than 30% and 60% of the tumors, respectively. High-level amplifications at 7q21.3–q22.1 and 19q13.2 and homozygous deletions at 1p33–p32.3, 1p22.1, 1q22, 3q27.2, 6p22.3, 6p21.31, 12q13.2, 17p13.2, 17q21.31 and 22q13.1 were identified. Especially, amplification of AKT2 was detected in two carcinomas and homozygous deletion of CDKN2C in other two cases. In 15 independent validation samples, we found that AKT2 (19q13.2) and MCM7 (7q22.1) were amplified in 6 and 9 cases, and CAMTA2 (17p13.2) and PFN1 (17p13.2) were homozygously deleted in 3 and 1 cases. AKT2 and MCM7 were overexpressed, and CAMTA2 and PFN1 were underexpressed in pancreatic cancer tissues than in morphologically normal operative margin tissues. Both GISTIC and Genomic Workbench software identified 22q13.1 containing APOBEC3A and APOBEC3B as the only homozygous deletion region. And the expression levels of APOBEC3A and APOBEC3B were significantly lower in tumor tissues than in morphologically normal operative margin tissues. Further validation showed that overexpression of PSCA was significantly associated with lymph node metastasis, and overexpression of HMGA2 was significantly associated with invasive depth of pancreatic cancer.

**Conclusion:**

These recurrent genomic changes may be useful for revealing the mechanism of pancreatic carcinogenesis and providing candidate biomarkers.

## Background

Pancreatic cancer is one of the most malignent cancers in the world with a 5-year survival rate of below 5% [1]. Up to now, there is not conventional treatment with a significant impact on the course of pancreatic cancer, so that the prognosis for patients still remains poor. Therefore, identification of the molecular changes underlying this cancer will lay the foundations for improving clinical management and outcomes.

Genomic instability is a characteristic feature of almost human tumors [2]. Copy number changes are frequently found in cancers, and are believed to contribute to the initiation and progression of tumors by amplification and activation of oncogenes or deletion-induced down-expression of tumor suppressor genes. Several previous studies have identified some recurrent chromosome alterations in pacreatic cancer, such as gains on 1q, chromosomes 2, 3 and 5, 7p, 8q, 11q, 12p, 14q, 17q, 19q and 20q, losses on chromosomes 1p, 3p, 6, 8p, 9p, 10q, 13q, 14q, 15q, 17p and 18q, and amplifications of FGFR1, HER2 and DcR3 [3], [4], [5], [6], [7], [8], [9]. However, the available information is still limited, especially for Chinese pancreatic cancer.

The present study identified common gains, losses, amplifications and homozygous deletions in pancreatic cancer. We further evaluated the protein expression level of the copy number-increased genes HMGA2 and PSCA.

## Materials and Methods

### Study Design

First, the genetic aberrations in pancreatic carcinomas were detected by using Agilent 44K Human Genome CGH microarray and common genomic changes were identified. Then, we validated the protein expression of HMGA2 and PSCA which were located in the common aberration chromosome regions in pancreactic cancer.

### Patients and Samples

Freshly resected tissues from 93 pancreatic carcinoma patients were collected by the Department of Pathology, Cancer Hospital, Chinese Academy of Medical Sciences, Beijing, China from 2006 to 2008. All the pancreatic cancer patients were treated with radical operation, and none of them received any treatment before surgery. Representative tumor regions were excised by experienced pathologists and immediately stored at −70°C until used. All the samples used in this study were residual specimens after diagnosis sampling. Every patient signed separate informed consent forms for sampling and molecular analysis. Clinical characteristics of patients used in the array CGH study are shown in Table 1. This study was approved by the Ethics Committee of Cancer Institute and Hospital, Peking Union Medical College, Chinese Academy of Medical Sciences (No. NCC2013B-30).

**Table 1:** Clinical Characteristics of 15 Patients Studied by Array CGH.

No.	Sex	Age	T	N	M	Grade	Pathology
01	Male	73	3	0	0	G2∼G3	Ductal adenocarcinoma
02	Male	60	3	1	0	G2∼G3	Ductal adenocarcinoma
03	Male	50	3	1	1	G1	Ductal adenocarcinoma
04	Female	42	3	1	0	G1	Ductal adenocarcinoma
05	Female	65	3	0	0	G1∼G2	Ductal adenocarcinoma
06	Male	64	3	1	0	G2	Ductal adenocarcinoma
07	Male	40	3	1	0	G2∼G3	Ductal adenocarcinoma
08	Female	40	3	1	1	G2	Ductal adenocarcinoma
09	Male	73	3	1	0	G3	Ductal adenocarcinoma
10	Male	62	3	1	0	G2∼G3	Ductal adenocarcinoma
11	Male	78	3	1	0	G2∼G3	Ductal adenocarcinoma
12	Male	43	3	1	0	G2∼G3	Ductal adenocarcinoma
13	Male	60	3	0	0	G3	Ductal adenocarcinoma
14	Female	74	3	1	1	G3	Ductal adenocarcinoma
15	Female	54	3	1	0	G3	Ductal adenocarcinoma

### Genomic DNA Extraction

Genomic DNA was isolated from tumor tissues using the Qiagen DNeasy Blood & Tissue Kit as described by the manufacturer (Qiagen, Hilden, Germany). Tumor cell content of all the samples was greater than 50% by HE staining.

### Array-based CGH

Array CGH experiments were performed using standard Agilent protocols (Agilent Technologies, Santa Clara, CA). Commercial human genomic DNA (PROMEGA, Warrington, UK) was used as reference. For each CGH hybridization, 500 ng of reference genomic DNA and the same amount of tumor DNA were digested with Alu I and RSA I restriction enzyme (PROMEGA, Warrington, UK). The digested reference DNA fragments were labeled with cyanine-3 dUTP and the tumor DNA with cyanine-5 dUTP (Agilent Technologies, Santa Clara, CA). After clean-up, reference and tumor DNA probes were mixed and hybridized onto Agilent 44K human genome CGH microarray (Agilent) for 40 h. Washing, scanning and data extraction procedures were performed following standard protocols.

### Microarray Data Analysis

Microarray data were analyzed using Agilent Genomic Workbench (Agilent Technologies, Santa Clara, CA) and BRB-arraytools (http://linus.nci.nih.gov/BRB-ArrayTools.html). Agilent Genomic Workbench was used to calculate log_2_^ratio^ for every probe and to identify genomic aberrations. Mean log_2_^ratio^ of all probes in a chromosome region between 0.25 and 0.75 was classified as genomic gain, >0.75 as high-level DNA amplification, <−0.25 as hemizygous loss, and <−0.75 as homozygous deletion. In pathway enrichment analysis, p-value is calculated for each pathway based on the null distribution obtained by a 1000-time random sampling method.

### Real-time PCR

The PCR reactions were performed in a total volume of 20 µl, including 10 µl of 2XPower SYBR Green PCR Master Mix (Applied Biosystems, Warrington, UK), 2 µl of cDNA/genomic DNA (5 ng/µl), and 1 µl of primer mix (10 µM each). The PCR amplification and detection were carried out in the ABI 7300 (Applied Biosystems, Warrington, UK) as follows: an initial denaturation at 95°C for 10 min; 45 cycles of 95°C for 15 s and 60°C for 1 min. The relative gene expression or relative copy number of the target gene was calculated using the comparative CT Method by normalized to an endogenous GAPDH. The relative to calibrator was given by the formula 2^−ΔΔCt^. ΔCT was calculated by subtracting the average GAPDH CT from the average CT of the gene of interest. The ratio defines the level of relative expression or relative copy number of the target gene to that of GAPDH. 2^−ΔΔCt^ >2,0 was set for a target amplification, and 2^−ΔΔCt^ <0.25 was set for a target homozygous deletion.

### Immunohistochemical staining

Formalin-fixed, paraffin-embedded pancreatic tumors were placed on the tissue microarray. For each case the cancer tissues were repeated for three times and adjacent morphologically normal tissues for two times. The slides were deparaffinized, rehydrated, immersed in 3% hydrogen peroxide solution for 10 min, heated in citrate buffer (pH 6) for 25 min at 95°C, and cooled for 60 min at room temperature. The slides were blocked by 10% normal goat serum for 30 min at 37°C and then incubated with mouse monoclonal antibody against HMGA2 (abcam, Cambridge, MA) and rabbit polyclonal antibody against PSCA (abcam, Cambridge, MA) overnight at 4°C. After being washed with PBS, the slides were incubated with biotinylated secondary antibody (diluted 1∶100) for 30 min at 37°C, followed by streptavidin-peroxidase (1∶100 dilution) incubation for 30 min at 37°C. Immunolabeling was visualized with a mixture of 3,3′-diaminobenzidine solution. Counterstaining was carried out with hematoxylin.

Expression level was determined on the basis of staining intensity and percentage of immunoreactive cells. Negative expression (score  = 0) was no or faint staining, or moderate to strong staining in <25% of cells. Weak expression (score  = 1) was a moderate or strong staining in 25% to 50% of cells. And strong expression (score  = 2) was >50% of the cells with strong staining.

### Statistical Analysis

Student's t-test and Chi square test were performed with the statistical software SPSS 15.0. The differences were judged as statistically significant when the corresponding two-sided *P* value were <.05.

## Results

### Gains and Losses in Pancreatic Carcinoma Detected by Array CGH

**Figure 1:**
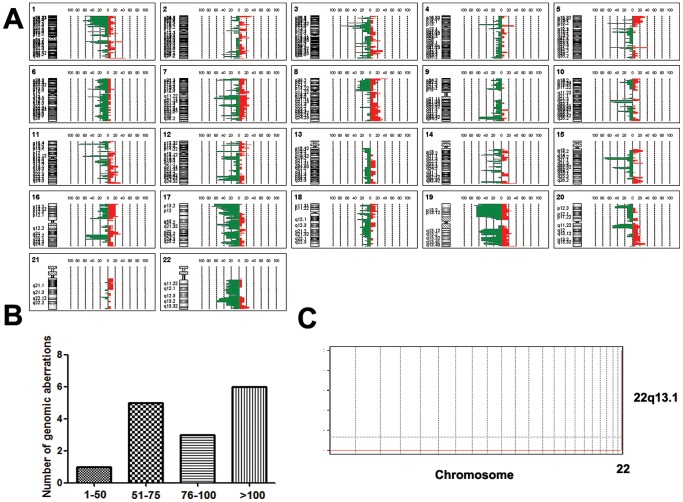
Genomic aberrations in pancreatic cancer. A. Genome-wide frequency plot of pancreatic cancer by array CGH analysis. Line on the right of 0%-axis, gain; line on the left of 0%-axis, loss. B. Numbers of aberrations in pancreatic cancer. X, number of aberrations; Y, number of cases. C. Gains and losses (HDs) identified by GISTIC.

Fifteen samples of pancreatic carcinoma were analyzed in this study and all of them had genomic changes (Range: 1 to 387). Sixteen gains and thirty-two losses were frequently detected (frequency of gain >30%, and loss >60%). The most frequent gains were 8p23.3 (41.7%), 1q44 (40%), 14q32.33 (40%), 19q13.43 (36.7%), 1q21.3 (36%) and 5q31.1–q31.2 (35.6%), and most common losses were 11p15.4 (70.7%), 15q15.1–q21.1 (70%), 3p21.31 (68.9%), 17p13.3–p13.2 (66.7%), 19p13.3–p13.2 (66.7%), 5p13.3 (63.3%), 11p11.2 (63.3%) and 19p13.3–p13.11 (63.3%). GISTIC analysis showed that copy number decrease of APOBEC3A (22q13.1) and APOBEC3B (22q13.1) was significant (Fig. 1 and Table 2).

**Table 2:** Genomic Gains and Losses in Pancreatic Cancer.

			region			
Change	No.	Cytoband	Start	End	Percent1 (%)	No. of probe
Gain	1	8p23.3	181530	1528274	41.7	14
	2	1q44	245415410	247179291	40.0	29
	3	14q32.33	105354886	106311914	40.0	8
	4	19q13.43	63558788	63784382	36.7	23
	5	1q21.3	150354126	151576549	36.0	41
	6	5q31.1–q31.2	134865707	136298888	35.6	24
	7	2p25.3	764887	3196999	33.3	18
	8	3q26.1	162699470	168905351	33.3	44
	9	4p13–p12	42742952	46671044	33.3	36
	10	5p15.33–p15.31	2209390	6426118	33.3	20
	11	8q24.23–q24.3	139224333	140752139	33.3	7
	12	8q24.3	144974801	145624565	33.3	25
	13	11q25	130772681	133432246	33.3	21
	14	12p13.2	10845519	11358635	33.3	17
	15	16q21	62462977	63621204	33.3	17
	16	20q13.32–q13.33	57782831	59579107	33.3	25
Loss	1	11p15.4	8754790	9967698	70.7	32
	2	15q15.1–q21.1	38644022	42843706	70.0	122
	3	3p21.31	46978276	49648485	68.9	98
	4	17p13.3–p13.2	769430	5382034	66.7	173
	5	19p13.3–p13.2	2323672	10394642	66.7	322
	6	5p13.3	32069173	32512980	63.3	16
	7	11p11.2	46490905	47989325	63.3	57
	8	19p13.2p13.11	10432688	19687095	63.3	437
	9	1p36.11–p32.3	26563174	51476264	62.2	95
	10	7q11.23	71858992	75893876	62.2	78
	11	1p36.33	1698756	2134018	60.0	11
	12	1q21.2–q21.3	148163183	149900117	60.0	75
	13	3p22.3	32516820	33442286	60.0	18
	14	4p14	39145576	40503807	60.0	28
	15	5q31.1	133588162	133774460	60.0	9
	16	7p22.1	5831281	6406280	60.0	16
	17	9q33.3	126479129	127679820	60.0	29
	18	9q33.3–q34.13	128491637	133095053	60.0	135
	19	10q21.3	69347447	70446758	60.0	31
	20	10q22.1	73557841	74435066	60.0	24
	21	12p11.21	31570586	32645521	60.0	16
	22	12q24.11–q24.13	108870045	111393102	60.0	67
	23	12q24.31	121290368	121666026	60.0	12
	24	16q21–q22.1	65103378	69280309	60.0	183
	25	16q22.3–q23.1	72893640	74235712	60.0	45
	26	17p13.1	6842796	8133829	60.0	92
	27	17q21.2–q21.31	37274288	39139633	60.0	95
	28	19p13.3	529533	557029	60.0	2
	29	19p13.3	633003	806290	60.0	11
	30	19q13.12	40386604	40896554	60.0	29
	31	19q13.12	41089222	42656912	60.0	55
	32	22q13.2	39505050	41219454	60.0	58

Note: 1: when two or more adjacent cytobands have copy number changes at a frequency above 30% (gain) and 60% (loss), the average frequency of these cytobands was calculated and listed.

### Amplifications and Homozygous Deletions in Pancreatic Carcinoma Detected by Array CGH

**Figure 2:**
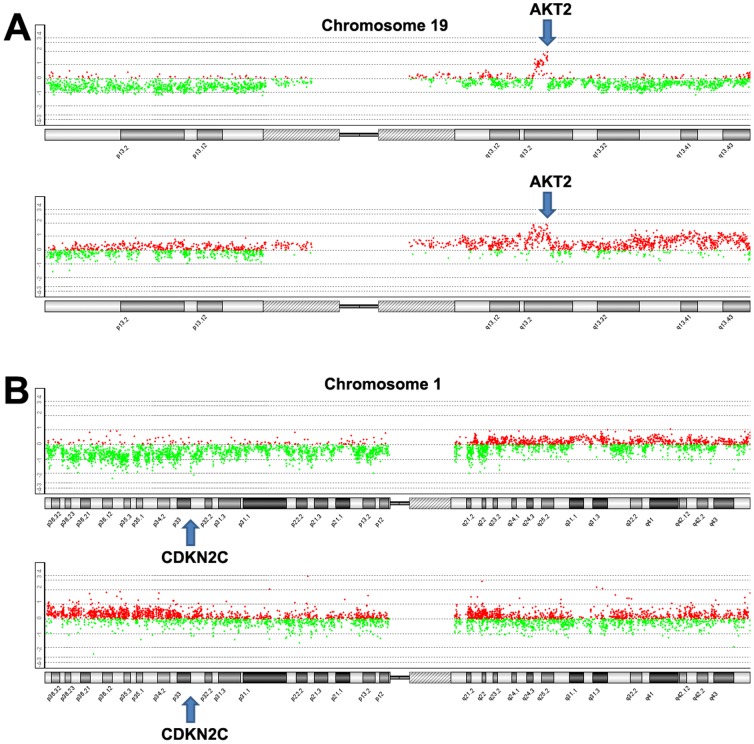
Amplification of AKT2 and homozygous deletion of CDKN2C in pancreatic cancer. A. amplification of AKT2. B. homozygous deletion of CDKN2C. Arrows indicate the position of AKT2 and CDKN2C.

**Figure 3:**
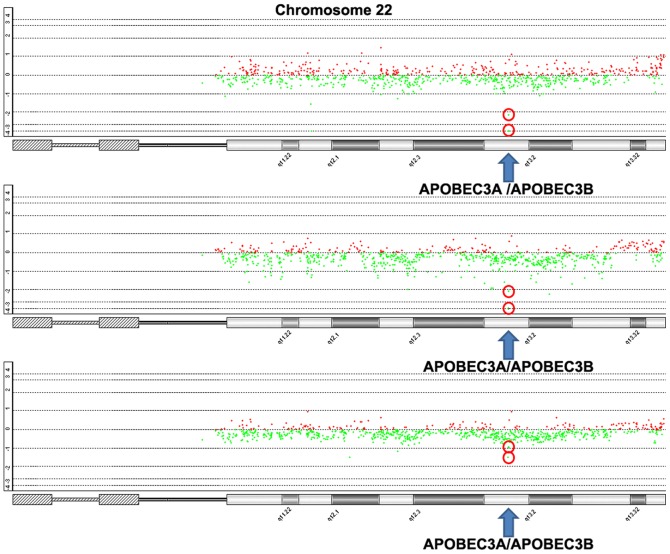
Homozygous deletion of APOBEC3A and APOBEC3B in pancreatic cancer. Cycles represent the probes of APOBEC3A and APOBEC3B.

High-level amplifications were detected at two chromosome regions including 7q21.3–q22.1 and 19q13.2. Homozygous deletions were identified in 1p33–p32.3, 1p22.1, 1q22, 3q27.2, 6p22.3, 6p21.31, 12q13.2, 17p13.2, 17q21.31 and 22q13.1 (Table 3). Especially, cancer gene AKT2 (19q13.2) was amplified in two carcinomas, and CDKN2C (1p33) was homozygously deleted in other two cases. (Fig. 2). By searching the COSMIC database, we found that amplification of AKT2 was associated with the increased sentitivity to the drug Z-LLNIe-CHO. More interestingly, homozygous deletion of 22q13.1 containing APOBEC3A and APOBEC3B was identified in both GISTIC and Agilent Genomic Workbench analysis (Fig. 3).

**Table 3:** High Level Amplifications and Homozygous Deletions in Pancreatic Cancer.

			Region			
Change	No.	Cytoband	Start	End	No. of cases	Gene
Amp	1	7q21.3–q22.1	97856949	101901147	2	BAIAP2L1, NPTX2, TMEM130, TRRAP, SMURF1, KPNA7, MYH16, ARPC1A, ARPC1B, PDAP1, BUD31, PTCD1, CPSF4, ATP5J2, ZNF789, ZNF394, ZKSCAN5, C7orf38, ZNF655, ZNF498, CYP3A5, CYP3A7, CYP3A4, CYP3A43, OR2AE1, TRIM4, GJC3, AZGP1, ZKSCAN1, ZSCAN21, ZNF3, COPS6, MCM7, AP4M1, TAF6, CNPY4, MBLAC1, C7orf59, C7orf43, GAL3ST4, GPC2, STAG3, GATS, PVRIG, SPDYE3, PMS2L1, PILRB, PILRA, ZCWPW1, MEPCE, C7orf47, LOC402573, TSC22D4, C7orf51, AGFG2, LRCH4, FBXO24, PCOLCE, MOSPD3, TFR2, ACTL6B, GNB2, GIGYF1, POP7, EPO, ZAN, EPHB4, SLC12A9, TRIP6, SRRT, UFSP1, ACHE, MUC17, TRIM56, SERPINE1, AP1S1, VGF, C7orf52, MOGAT3, PLOD3, ZNHIT1, CLDN15, FIS1, RABL5, EMID2, MYL10, CUX1, SH2B2, SPDYE6, PRKRIP1, ORAI2, ALKBH4, LRWD1, POLR2J
	2	19q13.2	45178101	45465385	2	PSMC4, ZNF546, ZNF780B, ZNF780A, MAP3K10, TTC9B, CNTD2, AKT2
HD	1	1p33–p32.3	51208696	51476264	2	CDKN2C, C1orf185, RNF11
	2	1p22.1	93077577	93587765	2	RPL5, SNORA66, FAM69A, MTF2, TMED5, CCDC18, DR1
	3	1q22	154178968	154245532	2	RXFP4, ARHGEF2, SSR2
	4	3q27.2	187138113	187416929	2	TRA2B, ETV5, DGKG
	5	6p22.3	16238624	16245913	3	MYLIP
	6	6p21.31	36466570	36671623	3	PXT1, KCTD20, STK38, SFRS3
	7	12q13.2	54785155	54799394	2	PA2G4, RPL41, ZC3H10
	8	17p13.2	4789213	4819488	2	RNF167, PFN1, ENO3, SPAG7, CAMTA2
	9	17q21.31	41566540	41624530	4	KIAA1267
	10	22q13.1	37689058	37715431	3	APOBEC3A, APOBEC3B

Note: Amp: amplifications. HD: homozygous deletions.

**Figure 4:**
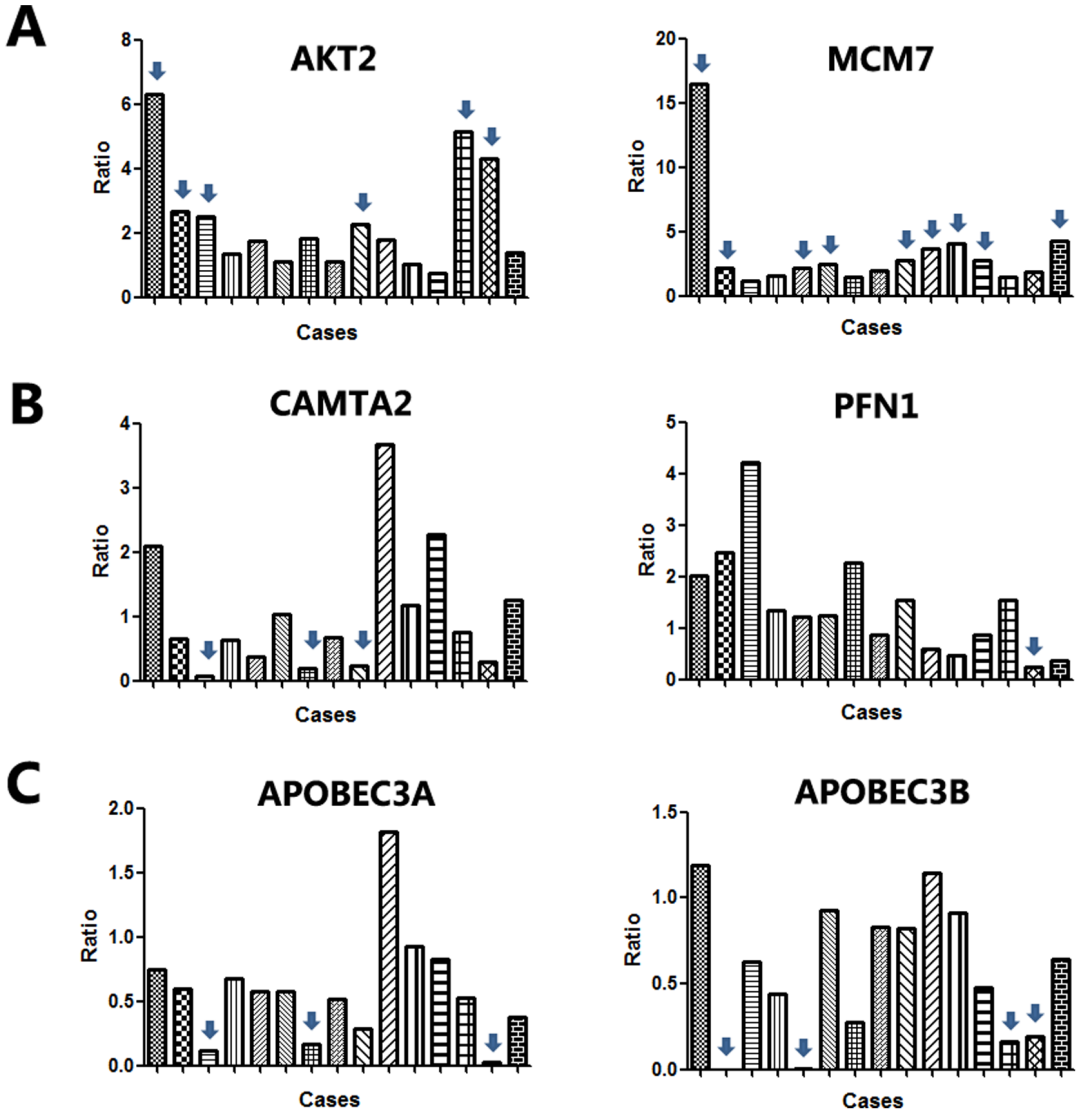
Validation of amplifications and homozygous deletions of candidate genes in independent pacreatic cancer tissues. A. amplication of AKT2 and MCM7. B. homozygous deletion of CAMTA2 and PFN1. C. homozygous deletion of APOBEC3A and APOBEC3B. Ratio = (Copy number of candidate gene in tumor tissues)/(Copy number of candidate gene in commercial human genomic DNA).

**Figure 5:**
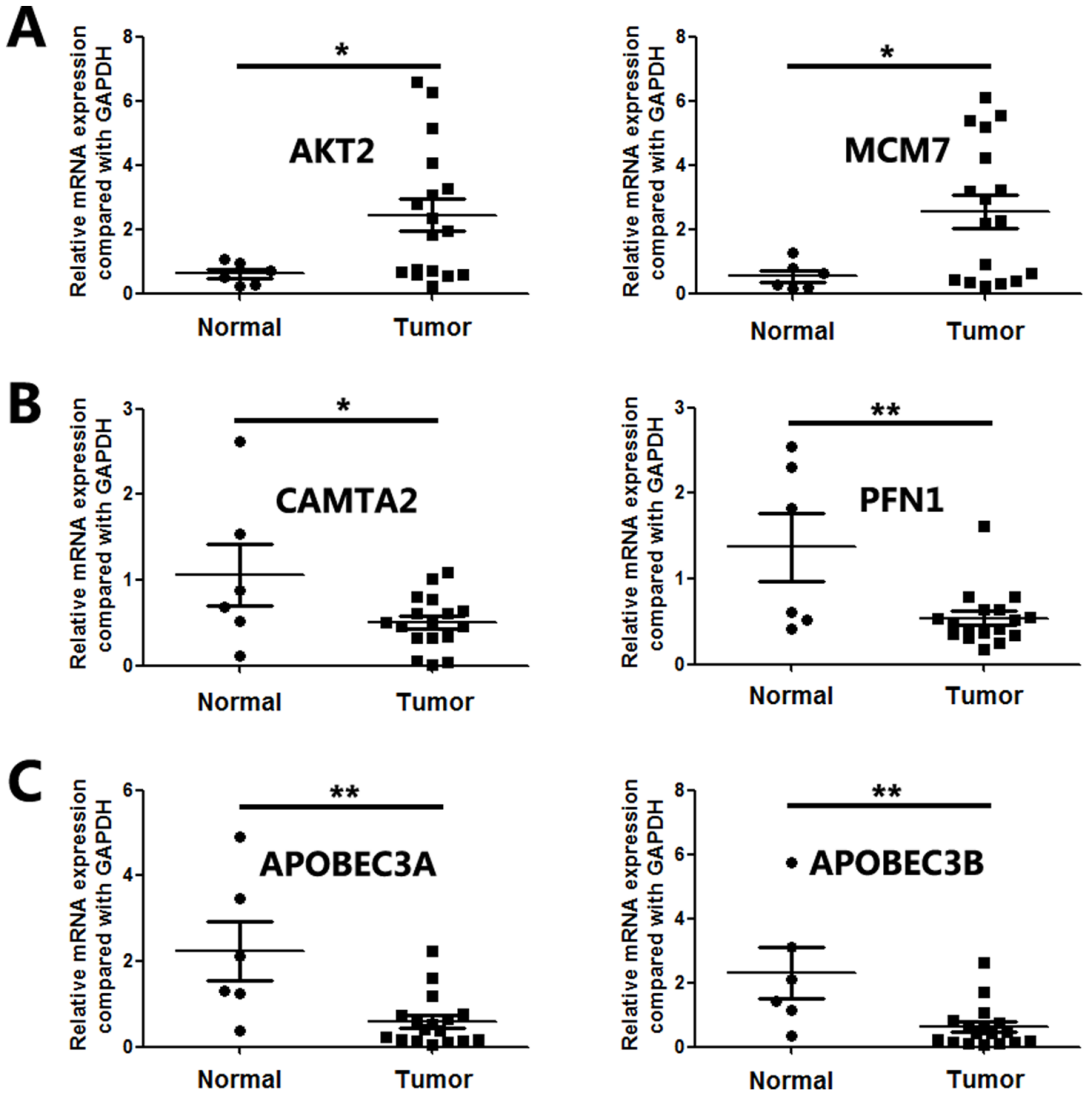
mRNA expression of candidate genes in pancreatic cancer as compared with that in morphologically normal operative margin tissues detected by using Real-time PCR. A. Overexpression of AKT2 and MCM7. B. Underexpression of CAMTA2 and PFN1. C. Underexpression of APOBEC3A and APOBEC3B.

We further selected the amplified genes AKT2 (19q13.2) and MCM7 (7q22.1) and homozygous deleted genes CAMTA2 (17p13.2) and PFN1 (17p13.2) for validation by real-time PCR. In 15 independent validation samples, amplifications of AKT2 and MCM7 were detected in 6 and 9 cases, and homozygous deletions of CAMTA2 and PFN1d in 3 and 1 cases, respectively (Fig. 4A and 4B). AKT2 and MCM7 were overexpressed, and CAMTA2 and PFN1 were underexpressed in pancreatic cancer tissues than in morphologically normal operative margin tissues (Fig. 5A and 5B).

In independent validation samples, APOBEC3A and APOBEC3B were homozygous deleted in 3 and 4 tumors, respectively (Fig. 4C). The mRNA expression levels of APOBEC3A and APOBEC3B in tumor tissues were significantly lower than in morphologically normal operative margin tissues (Fig. 5C)

### Pathways Enriched for Copy Number Alterations

Pathway enrichment analysis using KEGG database was applied to the CGH data. We found that two pathways enriched in genes with gain and that six pathways enriched in genes with loss. The genomic gains in pancreatic carcinoma changed the pathways of gamma-hexachlorocyclohexane degradation and oxidative phosphorylation. However, cyanoamino acid metabolism, glutathione metabolism, atrazine degradation, taurine and hypotaurine metabolism, arachidonic acid metabolism and parkinson's disease pathways were changed by the genomic losses (Table 4).

**Table 4:** Pathways Enriched in Array CGH Data.

Change	No.	Pathway	Description	No. of genes	*P* value
Gain	1	hsa00361	gamma-Hexachlorocyclohexane degradation	24	0.001
	2	hsa00190	Oxidative phosphorylation	112	0.004
Loss	1	hsa00460	Cyanoamino acid metabolism	9	0.001
	2	hsa00480	Glutathione metabolism	40	0.001
	3	hsa00791	Atrazine degradation	7	0.001
	4	hsa00430	Taurine and hypotaurine metabolism	10	0.002
	5	hsa00590	Arachidonic acid metabolism	55	0.005
	6	hsa05020	Parkinson's disease	15	0.007

### Validation of HMGA2 and PSCA in Pancreatic Cancer using Immunohistochemistry

**Figure 6:**
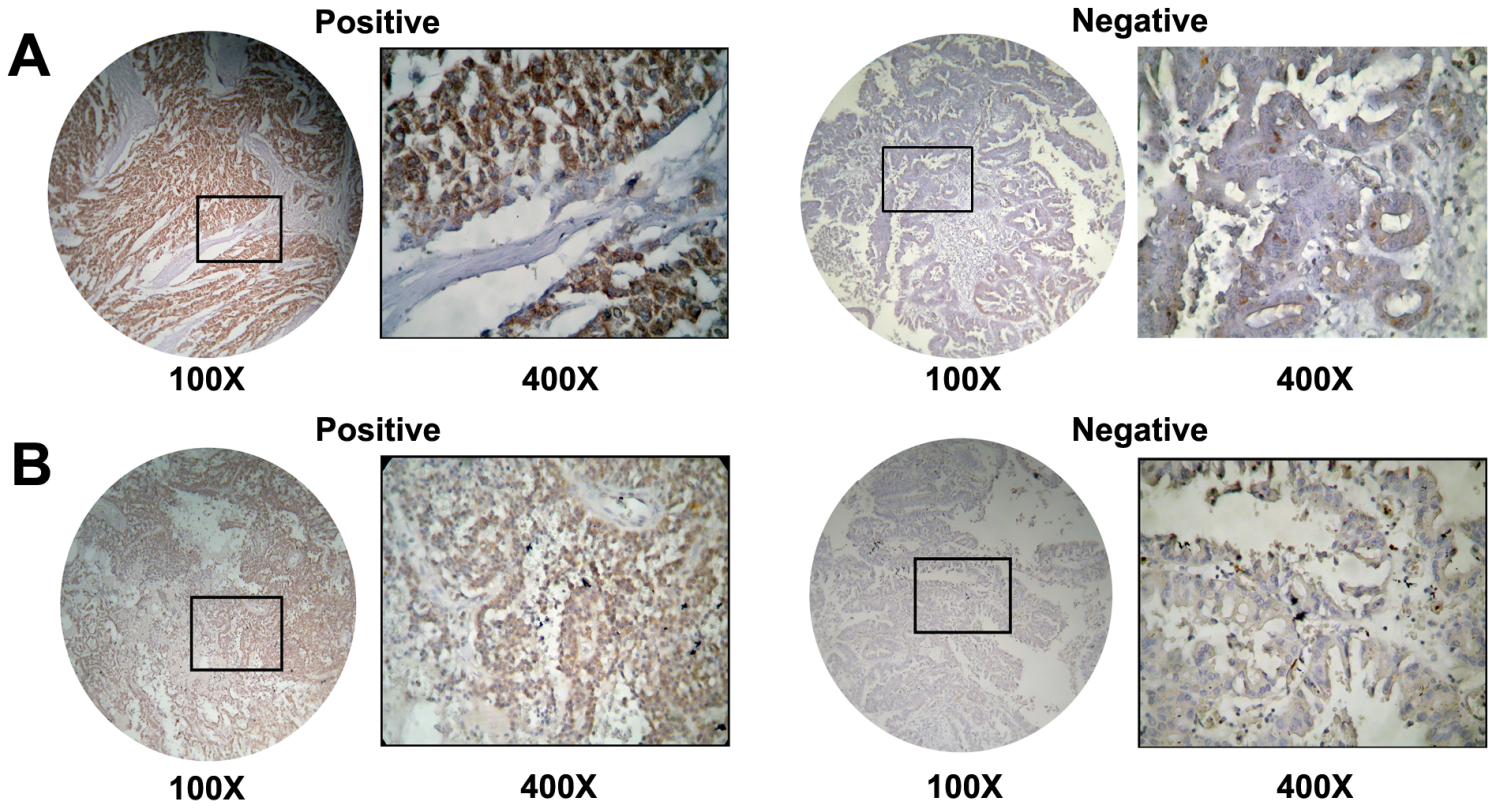
Representative immunohistochemistry results of HMGA2 and PSCA in pancreatic cancer as compared with those in morphologically normal operative margin tissues. A. Strong and negative expression of HMGA2. B. Strong and negative expression of PSCA.

Copy number increase of HMGA2 and PSCA was detected in one and four tumor, respectively. Because of their significant role in tumorigenesis [10], [11], [12], [13], we analyzed the protein expression of HMGA2 and PSCA using immunohistochemistry (IHC). The results showed that overexpression of HMGA2 and PSCA was detected in 76.7% and 65.0% of pancreatic cancer patients, respectively (Fig. 6). Further, overexpression of PSCA was significantly associated with lymph node metastasis (Table 5), and overexpression of HMGA2 was significantly associated with invasive depth of pancreatic cancer (Table 6).

**Table 5:** Association between PSCA Expression and Clinicopathological Characteristics of the Pancreatic Cancer.

	PSCA(n = 58)			P value
Clinical parameter	Negative	Positive	*χ^2^*	P value
Age			1.53	0.216
<60	9	14		
≥60	10	25		
Sex			0.83	0.362
Male	13	24		
Female	6	15		
pT			5.19	0.075
T1	2	0		
T2	1	6		
T3	16	33		
pN			4.37	0.037
N0	16	22		
N1	3	17		
pM			0.095	0.758
M0	16	34		
M1	3	5		
Grade			2.029	0.362
G1	3	5		
G2	6	20		
G3	10	14		

**Table 6:** Association between HMGA2 Expression and Clinicopathological Characteristics of the Pancreatic Cancer.

	HMGA2(n = 60)				
Clinical parameter	Negative	Weak positive	Strong positive	*χ^2^*	P value
Age				3.096	0.213
<60	5	14	6		
≥60	9	12	14		
Sex				0.754	0.686
Male	11	17	14		
Female	3	9	6		
pT				0.785	0.94
T1	0	1	1		
T2	2	3	2		
T3	12	22	17		
pN				13.062	0.001
N0	10	21	6		
N1	4	5	14		
pM				1.961	0.375
M0	10	23	17		
M1	4	3	3		
Grade				6.21	0.184
G1	1	6	2		
G2	7	10	9		
G3	6	10	9		

## Discussion

Genomic aberrations can contribute to the carcinogenesis and tumor progression. In order to identify DNA copy number changes in pancreatic cancer, we performed array-based comparative genomic hybridization and found that sixteen gains with frequency above 30% and thirty-two losses above 60%, with two high-level amplifications at 7q21.3–q22.1 and 19q13.2 and ten homozygous deletions at 1p33–p32.3, 1p22.1, 1q22, 3q27.2, 6p22.3, 6p21.31, 12q13.2, 17p13.2, 17q21.31 and 22q13.1. By comparing our results with CGH data presented in progenetix web site [14], [15], we found that most genomic aberrations were consistent. But there were still some differences. For example, loss of 9p was more frequent than loss of 9q in progenetix data, but the frequency of 9q loss was higher than 9p loss in our study. The gain of chromosome 7 was very common in progenetix data, but loss of this chromosome was more frequent in our data.

Significantly, cancer gene AKT2 was amplified in two pancreatic cancer patients, and cancer gene CDKN2C was homozygously deleted in other two cases. We validated the amplification of AKT2 and MCM7 (7q22.1) and homozygous deletion of CAMTA2 (17p13.2) and PFN1 (17p13.2) in pancreatic cancer, and further found that AKT2 and MCM7 were overexpressed, and CAMTA2 and PFN1 were underexpressed in pancreatic cancer as compared with those in morphologically normal operative margin tissues. These results suggested that genes including AKT2, MCM7, CAMTA2 and PFN1 might play important roles in pancreatic cancer. Homozygous deletion of CDKN2C has been found in myeloma, and copy number decrease of CDKN2C was significantly associated with a worse overall survival [16], [17], [18]. However, there was still little information about the role of CDKN2C in pancreatic cancer. Concerning the alteration of AKT2 in human malignancies, Miwa et al. have reported the amplification of AKT2 was in 3 of 12 pancreatic cancer cell lines and in 3 of 20 primary pancreatic carcinomas. Overexpression of AKT2 was also detected in the 3 cell lines with amplified AKT2 [19]. The up-regulation of AKT2 was correlated with the prognosis [20]. Tanno et al. found that active AKT promoted the invasiveness of pancreatic cancer cells through up-regulating IGF-IR expression [21]. RNAi simultaneously targeting AKT2 and K-ras could inhibite the pancreatic tumor growth [22]. Chen et al. demonstrated that AKT2 inhibition could abrogate gemcitabine-induced activation of AKT2 and NF-κB, and promote gemcitabine-induced PUMA upregulation, resulting in chemosensitization of pancreatic tumors to gencitabine [23]. Our results further verified the amplification of AKT2 in pancreatic cancer. By searching the COSMIC database, we also found that amplification of AKT2 was associated with the increased sentitivity to the drug Z-LLNIe-CHO. All these results suggested that amplification of AKT2 maybe develop into a biomarker to divide the pancreatic cancer patients into different subgroups for applying different therapy strategy. And in the future, whether the drug Z-LLNIe-CHO could be used to treat the pancreatic cancer patients with AKT2 amplification should be studied.

Interestingly, both GISTIC and Genomic Workbench Software identified 22q13.1 (containing APOBEC3A and APOBEC3B) as homozygous deletion region. Real-time PCR assay showed that APOBEC3A and APOBEC3B were underexpressed in pancreatic cancer tissues than in morphologically normal operative margin tissues. APOBEC enzymes function in innate immune responses, including those that target retroviruses, suggesting links between immunity, mutagenesis and viral infection in the process of cancer development. APOBEC3A could induce hypermutation of genomic DNA and DNA double strand breaks, and catalyze the transition from a healthy to a cancer genome [24], [25]. Pham et al. reported that APOBEC3A was expressed in keratinocytes, and up-regulated in skin cancer [26]. APOBEC3B was overexpressed in a majority of ovarian cancer cell lines and high grade primary ovarian cancers. Improtantly APOBEC3B expression was correlated with total mutaion load as well as elevated levels of transversion mutations [27]. Harris et al. reported that APOBEC3B accounted for up to half of the mutational load in breast carcinomas expressing this enzyme [28]. In other cancers including bladder, cervix, lung and head and neck, APOBEC3B was also upregulated and its preferred target sequence was frequently mutated and clustered [29]. Deletion of APOBEC3B attenuated HBV clearance, and resulted in HBV infection and increased risk for developing hepatocellular carcinoma [30]. Deletion of APOBEC3 was also associated with breast cancer risk among women of European ancestry [31]. Homozygous deletion of APOBEC3B was significantly associated with unfavorable outcomes for HIV-1 acquisition and progression to AIDS [32]. It will be interesting to investigate the role of homozygous deletion of APOBEC3A and APOBEC3B in the pancreatic carcinogenesis.

HMGA2 and PSCA have been reported to be associated with pancreatic cancer. Piscuoglio et al. showed that the percentage of tumor cells with HMGA2 and HMGA1 nuclear immunoreactivity correlated positively with increasing malignancy grade and lymph node metastasis [33], [34]. And HMGA2 maintained oncogenic RAS-induced epithelial-mesenchymal transition in human pancreatic cancer cells [35]. Our study revealed that gains of HMGA2 and PSCA were detected in one and four pancreatic carcinomas, respectively. In IHC assay, overexpression of HMGA2 was detected in 76.7% and that of PSCA in 65.0% of tumors. And overexpression of PSCA was significantly associated with lymph node metastasis, and overexpression of HMGA2 was significantly associated with invasive depth of pancreatic cancer.

Overall, our study identified multiple copy number-altered chromosome regions in pancreatic cancer. These findings provide important insights into the molecular alterations associated with pancreatic tumorigenesis. Further studies should be conducted to explore the possible tumorigenic roles of these copy number changed genes.
